# Supplementary material for: Enteric pathogens in soil adjacent to public waste bins in Maputo, Mozambique offers potential for environmental surveillance
Source: PLOS Glob Public Health. 2026 Aug 18;6(8):e0005998. doi: 10.1371/journal.pgph.0005998 (PMC13485064; doi:10.1371/journal.pgph.0005998)
Supplement: Supplementary file 1 [file pgph.0005998.s001.docx]

S1 Appendix to:

Enteric Pathogens in Soil Adjacent to Public Waste Bins in Maputo, Mozambique Offers Potential for Environmental Surveillance

Jack Dalton^1,2^, Gouthami Rao^2^, Marcia Chiluvane^3^, Victoria Cumbane^3^, David Holcomb^2^, Erin Kowalsky^4^, Amanda Lai^2^, Elly Mataveia^3^, Vanessa Monteiro^3^, Edna Viegas^3^, Joe Brown^2^, Drew Capone^5*^,

1. School of Civil Engineering, Faculty of Engineering and Physical Science, University of Leeds, Leeds, England, United Kingdom
2. Department of Environmental Sciences and Engineering, University of North Carolina at Chapel Hill, Chapel Hill, North Carolina, United States of America
3. Centro de Investigação e Treino em Saúde da Polana Caniço (CISPOC), Instituto Nacional de Saúde, Maputo, Mozambique
4. School of Medicine, Emory University, Atlanta, Georgia, United States of America
5. Department of Environmental and Occupational Health, School of Public Health-Bloomington, Indiana University, Bloomington, Indiana, United States of America

*Corresponding Author: Drew Capone, dscapone@iu.edu

**Supporting Information Available**

Contents

[S1 Appendix Table A: Summary Statistics for Unique Enteric Pathogen Detections by Site Location	2](#_Toc236488499)

[S1 Appendix Table B Target Percent Detection with 95% Confidence Interval	2](#_Toc236488500)

[S1 Appendix Table C. Assays	3](#_Toc236488501)

[S1 Appendix Table D. Standard Curve	8](#_Toc236488502)

[S1 Appendix Table E. MIQE Checklist	10](#_Toc236488503)

[S1 Appendix Checklist A. Inclusivity in global research	13](#_Toc236488504)

| S1 Appendix Table A: Summary Statistics for Unique Enteric Pathogen Detections by Site Location | | | | | |
| --- | --- | --- | --- | --- | --- |
| *Site Location* | *Mean* | *Median* | *Std Dev* | *Min* | *Max* |
| Basin farm area (n=19) | 3.80 | 2.00 | 4.77 | 0 | 20 |
| Non-sewered area (n=32) | 7.90 | 8.50 | 3.04 | 1 | 12 |
| Sewered system area (n=20) | 4.60 | 4.50 | 2.48 | 1 | 9 |
| 150-Meter Buffer Area (n=10) | 10.5 | 11.00 | 3.31 | 4 | 15 |

## S1 Appendix Table B Target Percent Detection with 95% Confidence Interval

| **Pathogen** | **Percent Detection (%)** | **Lower CI** | **Upper CI** |
| --- | --- | --- | --- |
| **Bacteria** |  |  |  |
| Aeromonas | 63.0 | 52.3 | 72.7 |
| C. difficile | 5.4 | 2.0 | 12.8 |
| Campylobacter jejuni | 14.1 | 8.0 | 23.3 |
| Vibrio spp. | 51.1 | 40.5 | 61.6 |
| E. coli | 33.7 | 24.4 | 44.4 |
| EAEC | 33.7 | 24.4 | 44.4 |
| EIEC | 23.9 | 15.9 | 34.1 |
| EPEC | 28.3 | 19.6 | 38.8 |
| ETEC | 30.4 | 21.5 | 41.0 |
| Helicobacter pylori | 6.5 | 2.7 | 14.2 |
| Leptospira | 4.3 | 1.4 | 11.4 |
| Mycobacterium tuberculosis | 12.0 | 6.4 | 20.8 |
| Salmonella | 18.5 | 11.4 | 28.2 |
| STEC | 9.8 | 4.8 | 18.2 |
| **Helminth** |  |  |  |
| Ancylostoma duodenale | 4.3 | 1.4 | 11.4 |
| Ascaris lumbricoides | 26.1 | 17.7 | 36.5 |
| Necator americanus | 4.3 | 1.4 | 11.4 |
| Toxocara canis/cati | 3.3 | 0.8 | 9.9 |
| Trichuris trichiura | 14.1 | 8.0 | 23.3 |
| **Protozoa** |  |  |  |
| Cryptosporidium spp. | 53.3 | 42.6 | 63.6 |
| Entamoeba histolytica | 6.5 | 2.7 | 14.2 |
| Giardia spp. | 19.6 | 12.3 | 29.4 |
| **Virus** |  |  |  |
| Adenovirus 40/41 | 13.0 | 7.2 | 22.1 |
| Astrovirus | 7.6 | 3.4 | 15.6 |
| HIV | 1.1 | 0.1 | 6.8 |
| Norovirus | 3.3 | 0.8 | 9.9 |
| Rotavirus | 9.8 | 4.8 | 18.2 |
| Sapovirus | 9.8 | 4.8 | 18.2 |
| SARS-CoV-2 | 6.5 | 2.7 | 14.2 |
| Zika | 2.2 | 0.4 | 8.4 |
| **Fecal Source Trackers & AMR** |  |  |  |
| Human mtDNA | 82.6 | 73.0 | 89.4 |
| Avian bacteroides | 31.5 | 22.5 | 42.2 |
| Chicken mtDNA | 63.0 | 52.3 | 72.7 |
| Canine mtDNA | 53.3 | 42.6 | 63.6 |
| Class 1 Integron-Integrase Gene | 80.4 | 70.6 | 87.7 |

## S1 Appendix Table C. Assays

| # | Organism Name | Gene Name | Oligonucleotide Primer or Probe Sequence  (5’ – 3’) | Ref |
| --- | --- | --- | --- | --- |
| 1 | adenovirus 40-41 | Fiber gene | Fwd: AACTTTCTCTCTTAATAGACGCC  Rev: AGGGGGCTAGAAAACAAAA  Probe: CTGACACGGGCACTCT | ^12^ |
| 2 | astrovirus | Capsid | Fwd: CAGTTGCTTGCTGCGTTCA  Rev: CTTGCTAGCCATCACACTTCT  Probe: CACAGAAGAGCAACTCCATCGC | ^12^ |
| 3 | norovirus GI | ORF1-2 | Fwd: CGCTGGATGCGATTCCATGA  Rev: CTTAGACGCCATCATCATTTAC  Probe: TGGACAGGAGATCGC | ^12^ |
| 4 | norovirus GII | ORF1-2 | Fwd: CAAGAACCTATGTTTAGATGGATGAG  Rev: TCGACGCCATCTTCATTCACA  Probe: TGGGAGGGCGATCGCAATCT | ^12^ |
| 5 | rotavirus | NSP3 | Fwd: ACCATCTTCACGTAACCCTCTATGAG  Rev: GGTCACATAACGCCCCTATAGC  Probe: AGTTAAAAGCTAACACTGTCAAA | ^12^ |
| 6 | sapovirus | RdRp | Fwd 1: GACCAGGCTCTCGCCACCTAC  Fwd 2: TTGGCCCTCGCCACCTAC  Rev: CCCTCCATTTCAAACACTA  Probe: CCGCCTATGAACCA | ^12^ |
| 7 | EAEC | aaiC | Fwd: ATTGTCCTCAGGCATTTCAC  Rev: ACGACACCCCTGATAAACAA  Probe: TAGTGCATACTCATCATTTAAG | ^12^ |
| 8 | EAEC | aatA | Fwd: CTGGCGAAAGACTGTATCAT  Rev: TTTTGCTTCATAAGCCGATAGA  Probe: TGGTTCTCATCTATTACAGACAGC | ^12^ |
| 9 | STEC | *stx1* | Fwd: ACTTCTCGACTGCAAAGACGTATG  Rev: ACAAATTATCCCCTGWGCCACTATC  Probe: CTCTGCAATAGGTACTCCA | ^12^ |
| 10 | STEC | *stx2* | Fwd: CCACATCGGTGTCTGTTATTAACC  Rev: GGTCAAAACGCGCCTGATAG  Probe: TTGCTGTGGATATACGAGG | ^12^ |
| 11 | *E. coli* O157:H7 | *rfbE* | Fwd: TTTCACACTTATTGGATGGTCTCAA  Rev: CGATGAGTTTATCTGCAAGGTGAT  Probe: CTCTCTTTCCTCTGCGGTCCT | ^12^ |
| 12 | EPEC | eae | Fwd: CATTGATCAGGATTTTTCTGGTGATA  Rev: CTCATGCGGAAATAGCCGTTA  Probe: ATACTGGCGAGACTATTTCAA | ^12^ |
| 13 | EPEC | bfpA | Fwd: TGGTGCTTGCGCTTGCT  Rev: CGTTGCGCTCATTACTTCTG  Probe: CAGTCTGCGTCTGATTCCAA | ^12^ |
| 14 | ETEC | LT | Fwd: TTCCCACCGGATCACCAA  Rev: CAACCTTGTGGTGCATGATGA  Probe: CTTGGAGAGAAGAACCCT | ^12^ |
| 15 | ETEC | STh | Fwd: GCTAAACCAGCAGGGTCTTCAAAA  Rev: CCCGGTACAAGCAGGATTACAACA  Probe: TGGTCCTGAAAGCATGAA | ^12^ |
| 16 | ETEC | STp | Fwd: TGAATCACTTGACTCTTCAAAA  Rev: GGCAGGATTACAACAAAGTT  Probe: TGAACAACACATTTTACTGCT | ^12^ |
| 17 | *Shigella*/EIEC | *ipaH* | Fwd: CCTTTTCCGCGTTCCTTGA  Rev: CGGAATCCGGAGGTATTGC  Probe: CGCCTTTCCGATACCGTCTCTGCA | ^12^ |
| 18 | *Campylobacter jejuni/coli* | cadF | Fwd: CTGCTAAACCATAGAAATAAAATTTCTCAC  Rev: CTTTGAAGGTAATTTAGATATGGATAATCG  Probe: CATTTTGACGATTTTTGGCTTGA | ^12^ |
| 19 | *Salmonella enterica* | *ttr* | Fwd: CTCACCAGGAGATTACAACATGG  Rev: AGCTCAGACCAAAAGTGACCATC  Probe: CACCGACGGCGAGACCGACTTT | ^12^ |
| 20 | *Vibrio cholerae* | *hylA* | Fwd: ATCGTCAGTTTGGAGCCAGT  Rev: TCGATGCGTTAAACACGAAG  Probe: ACCGATGCGATTGCCCAA | ^12^ |
| 21 | *Clostridium difficile* | tcdB | Fwd: GGTATTACCTAATGCTCCAAATAG  Rev: TTTGTGCCATCATTTTCTAAGC  Probe: CCTGGTGTCCATCCTGTTTC | ^12^ |
| 22 | *Aeromonas* spp. | Aerolysin | Fwd: TTCGTTACCAGTGGGACAAG  Rev: CCGGCAAACTGGCTCTCG  Probe: CAGTTCCAGTCCCACCACTT | ^12^ |
| 23 | *Helicobacter pylori* | ureC | Fwd: GACACCAGAAAAAGCGGCTA  Rev: AGCGCATGTCTTCGGTTAAA  Probe: TCACTAAAGCGTTTTCTACC | ^12^ |
| 24 | *Cryptosporidium* spp. | 18S rRNA | Fwd: GGGTTGTATTTATTAGATAAAGAACCA  Rev: AGGCCAATACCCTACCGTCT  Probe: TGACATATCATTCAAGTTTCTGAC | ^12^ |
| 25 | *Giardia* spp. | 18S rRNA | Fwd: GACGGCTCAGGACAACGGTT  Rev: TTGCCAGCGGTGTCCG  Probe: CCCGCGGCGGTCCCTGCTAG | ^12^ |
| 26 | *Entamoeba histolytica* | 18S rRNA | Fwd: ATTGTCGTGGCATCCTAACTCA  Rev: GCGGACGGCTCATTATAACA  Probe: TCATTGAATGAATTGGCCATTT | ^12^ |
| 27 | *Ascaris lumbricoides* | *ITS1* | Fwd: GCCACATAGTAAATTGCACACAAAT  Rev: GCCTTTCTAACAAGCCCAACAT  Probe: TTGGCGGACAATTGCATGCGAT | ^12^ |
| 28 | *Trichuris trichiura* | 18S rRNA | Fwd: TTGAAACGACTTGCTCATCAACTT  Rev: CTGATTCTCCGTTAACCGTTGTC  Probe: CGATGGTACGCTACGTGCTTACCATGG | ^12^ |
| 29 | *Ancylostoma duodenale* | *ITS2* | Fwd: GAATGACAGCAAACTCGTTGTTG  Rev: ATACTAGCCACTGCCGAAACGT  Probe: ATCGTTTACCGACTTTAG | ^12^ |
| 30 | *Necator americanus* | *ITS2* | Fwd: CTGTTTGTCGAACGGTACTTGC  Rev: ATAACAGCGTGCACATGTTGC  Probe: CTGTACTACGCATTGTATAC | ^12^ |
| 31 | BRSV | BRSV | Fwd: GCAATGCTGCAGGACTAGGTATAAT  Rev: ACACTGTAATTGATGACCCCATTCT  Probe: ACCAAGACTTGTATGATGCTGCCAAAGCA | ^19^ |
| 32 | BHV | BHV | Fwd: GAGCAAAGCCCCGCCGAAGGA  Rev: TACGAACAGCAGCACGGGCGG  Probe: GAACCTGCCCACGCGCTGAAAC | ^20^ |
| 33 | pan-16S | 16S | Fwd: ATGGYTGTCGTCAGCT  Rev: ACGGGCGGTGTGTAC  Probe: CAACGAGCGCAACCC | ^21^ |
| 34 | *Mycobacterium tuberculosis* | IS6110 | Fwd: GGGTAGCAGACCTCACCTATG  Rev: AGCGTAGGCGTCGGTGA  Probe: TCGCCTACGTGGCCTTT | ^22^ |
| 35 | Toxocara spp. | *cox1* | Fwd: AAAATAGCCAAATCCACACTACTACCA  Rev: GGTGTGGGACTAGTTGAACTGTGTA  Probe: CCCCATAGTCCTCAAAG | ^5^ |
| 36 | Hs99999901_s1 | 18s | Thermofisher Internal Control | |
| 37 | SARS-CoV-2 | N1 | Fwd: GACCCCAAAATCAGCGAAAT  Rev: TCTGGTTACTGCCAGTTGAATCTG  Probe: ACCCCGCATTACGTTTGGTGGACC | ^6^ |
| 38 | avian 16s | Av4143 | Fwd: TGCAAGTCGAACGAGGATTTCT  Rev: TCACCTTGGTAGGCCGTTACC  Probe: AGGTGGTTTTGCTATCGCTTT | ^7^ |
| 39 | HIV proviral DNA | capsid p24 | Fwd: TGGGTAAAAGTAGTAGAAGAGAAGGCTTT  Rev: GCTATGTCACTTCCCCTTGGTTCT  Probe: TCAGCATTATCAGAAGGAG | ^8^ |
| 40 | *Plasmodium* spp. |  | Fwd: GTTAAGGGAGTGAAGACGATCAGA  Rev: AACCCAAAGACTTTGATTTCTCATAA  Probe: CCGTCGTAATCTTAACCATAAACTATGCCGACT | ^23^ |
| 41 | Leptospira spp. | *lipL32* | Fwd: AAGCATTACCGCTTGTGGTG  Rev: GAACTCCCATTTCAGCGATT  Probe: AAAGCCAGGACAAGCGCCG | ^10^ |
| 42 | poultry mtDNA | *cytb* | Fwd: AAATCCCACCCCCTACTAAAAATAAT  Rev: CAGATGAAGAAGAATGAGGCG  Probe: ACAACTCCCTAATCGACCT | ^11^ |
| 43 | EAEC2 | aggR | Fwd: GCAATCAGATTAAGCAGCGATACA  Rev: TTCGGACAACTGCAAGCATC  Probe: AAGACGCCTAAAGGATGCCC | ^12^ |
| 44 | hCYTB484 (human mtDNA) | cytochrome *b* | Fwd: CAATGAATCTGAGGAGGCTAC  Rev: CGTGCAAGAATAGGAGGTG  Probe: ACCCTCACACGATTCTTTACCTTTCACT | ^13^ |
| 45 | intI1 | intI1 | Fwd: GCCTTGATGTTACCCGAGAG  Rev: GATCGGTCGAATGCGTGT  Probe: ATTCCTCGTGGTTCTGGGTTTT | ^24^ |
| 46 | zika | ZIKV | Fwd: CCGCTGCCCAACACAAG  Rev: CCACTAACGTTCTTTTGCAGACAT  Probe: AGCCTACCTTGACAAGCAGTCAGACACTCAA | ^25^ |
| 47 | canine mtDNA | NADH subunit 5 marker | Fwd: GGCATGCCTTTCCTTACAGGATTC  Rev: GGGATGTGGCAACGAGTGTAATTATG  Probe: TCATCGAGTCCGCTAACACGTCGAAT | ^26^ |
| 48 | hepatitis G | hepG | Fwd: CGGCCAAAAGGTGGTGGATG  Rev: CGACGAGCCTGACGTCGGG  Probe: AGGTCCCTCTGGCGCTTGTGGCGAG | ^12^ |

Cycling conditions with a 1C/s ramp rate between all steps: 45°C for 20 minutes, 95°C for 10 minutes, then 50 cycles of 95°C for 15 seconds and 60°C for 1 minute.

## S1 Appendix Table D. Standard Curve

| Target | Intercept | Slope | R^2^ | Efficiency (%) | Low.95 (gc/rxn) | LOD (gc/rxn) | LOQ (gc/rxn) |
| --- | --- | --- | --- | --- | --- | --- | --- |
| 16S pan | 31.6 | -1.890 | 0.8360 | 116 | 0.1 | NA | 0.1 |
| 18s (manufacturer control) | NA |  |  |  |  |  |  |
| adenovirus 40/41 | 34.4 | -3.313 | 0.9968 | 100.4 | 0.0 | NA | 0.001 |
| *Aeromonas* spp. | 35.3 | -3.383 | 0.9987 | 97.5 | 10.0 | 6.98 | 6.98 |
| *Ancylostoma duodenale* | 36.6 | -3.359 | 0.9973 | 98.5 | 3.2 | 1.52 | 212 |
| *Ascaris lumbricoides* | 35.8 | -3.407 | 0.9987 | 96.6 | 100.0 | 8.66 | 240 |
| astrovirus | 34.2 | -3.368 | 0.9971 | 98.1 | 10.0 | 3.63 | 152 |
| BHV | 36.7 | -3.409 | 0.9992 | 96.5 | 100.0 | 5.38 | 65.0 |
| BRSV | 34.4 | -3.261 | 0.9956 | 102.6 | 3.2 | 5.13 | 5.13 |
| *Clostridium difficile* | 34.5 | -3.367 | 0.9983 | 98.1 | 10.0 | 1.78 | 12.0 |
| *Campylobacter jejuni/coli* | 35.5 | -3.408 | 0.9986 | 96.5 | 10.0 | 6.48 | 500 |
| *Cryptosporidium* spp. | 36.0 | -3.407 | 0.9987 | 96.6 | 10.0 | 6.50 | Inf |
| EAEC_(*aaiC*) | 35.9 | -3.436 | 0.9989 | 95.4 | 10.0 | 7.44 | 7.44 |
| EAEC_(*aatA*) | 35.0 | -3.404 | 0.9961 | 96.7 | 10.0 | 5.32 | 2832 |
| EIEC / *Shigella* | 35.4 | -3.425 | 0.9974 | 95.9 | 10.0 | 3.49 | 311 |
| EPEC (*bfpA*) | 36.1 | -3.490 | 0.9979 | 93.4 | 10.0 | 9.54 | Inf |
| EPEC (*eae*) | 34.4 | -3.354 | 0.9966 | 98.7 | 3.2 | 3.04 | 6.00 |
| ETEC (*LT*) | 35.8 | -3.399 | 0.9991 | 96.9 | 3.2 | 3.78 | 301 |
| ETEC (*STh*) | 35.4 | -3.408 | 0.9985 | 96.5 | 3.2 | 2.40 | 93 |
| ETEC (*STp*) | 35.9 | -3.187 | 0.9892 | 106.0 | 10.0 | NA | 1159 |
| E. coli O157:H7 | 35.1 | -3.368 | 0.9979 | 98.1 | 3.2 | 2.70 | 849 |
| *Entamoeba histolytica* | 36.3 | -3.442 | 0.9975 | 95.2 | 100.0 | 7.17 | 108 |
| *Giardia* spp. | 35.1 | -3.365 | 0.9986 | 98.2 | 10.0 | 6.44 | 2250 |
| HIV | 36.6 | -3.410 | 0.9985 | 96.4 | 10.0 | 4.83 | 3808 |
| *Helicobacter pylori* | 35.4 | -3.468 | 0.9977 | 94.3 | 10.0 | 4.30 | 689 |
| Human mtDNA | 34.9 | -3.354 | 0.9974 | 98.7 | 3.2 | 1.87 | 165 |
| *Necator americanus* | 36.2 | -3.437 | 0.9986 | 95.4 | 3.2 | 1.94 | 1596 |
| norovirus GI | 33.5 | -3.325 | 0.9991 | 99.9 | 10.0 | 3.49 | 12 |
| norovirus GII | 36.2 | -3.342 | 0.9974 | 99.2 | 3.2 | 3.33 | 131 |
| rotavirus | 35.6 | -3.395 | 0.9967 | 97.0 | 10.0 | 3.76 | 62 |
| SARS-COV-2 (N1) | 35.7 | -3.373 | 0.9977 | 97.9 | 10.0 | 4.48 | Inf |
| STEC (*stx1*) | 35.8 | -3.431 | 0.9989 | 95.7 | 3.2 | 3.78 | 540 |
| STEC (*stx2*) | 33.3 | -2.950 | 0.9620 | 118.3 | 0.1 | NA | 0.1 |
| *Salmonella* spp. (*ttr*) | 36.0 | -3.428 | 0.9979 | 95.8 | 100.0 | 9.18 | 5604 |
| sapovirus | 37.3 | -3.190 | 0.9953 | 105.8 | 10.0 | 9.59 | 5336 |
| *Trichuris trichiura* | 34.2 | -3.307 | 0.9980 | 100.6 | 1.0 | 0.50 | 0.501 |
| *Vibrio cholerae (hylA)* | 32.4 | -3.254 | 0.9876 | 102.9 | 10.0 | 2.79 | 914 |
| Avian 16s | 35.8 | -3.403 | 0.9961 | 96.7 | 10.0 | 12.91 | 1083 |
| Canine mtDNA | 35.7 | -3.246 | 0.9934 | 103.3 | 10.0 | 6.95 | 336 |
| EAEC (*aggR*) | 35.6 | -3.411 | 0.9989 | 96.4 | 3.2 | 3.35 | 294 |
| Hepatitis G | 35.6 | -3.344 | 0.9953 | 99.1 | 10.0 | NA | 2.00 |
| intl1 | 33.6 | -3.412 | 0.9978 | 96.4 | 10.0 | 2.06 | 4.00 |
| *Leptospira* spp. | 37.5 | -3.363 | 0.9977 | 98.3 | 3.2 | 1.62 | 375 |
| *Plasmodium* spp.* | 36.5 | -3.404 | 0.9968 | 96.7 | 3.2 | 1.99 | 78 |
| *Mycobacterium tuberculosis* | 36.9 | -3.367 | 0.9970 | 98.2 | 10.0 | 4.95 | 4.95 |
| Poultry mtDNA | 36.0 | -3.411 | 0.9975 | 96.4 | 10.0 | 16.18 | 9314 |
| *Toxocara* spp. | 35.9 | -3.420 | 0.9982 | 96.1 | 100.0 | 12.26 | 58.0 |
| zika | 33.4 | -3.239 | 0.9950 | 103.6 | 10.0 | 6.94 | 6.94 |

*Excluded from analyses due to apparent cross-reactivity. Low.95 is the lowest standard with at least 95% positive detection. LOD is the 95% limit of detection as determined by probit modeling. LOQ is the limit of quantification as determined by decay modeling, using the user-selected CV threshold of 0.35. TAC reaction wells have a volume of 1.5 µL, comprising 0.6 µL of template and 0.9 µL of mastermix. Gene copies per reaction (gc/rxn).

## S1 Appendix Table E. MIQE Checklist

| **ITEM TO CHECK** | **IMPORTANCE** | **CHECKLIST** |
| --- | --- | --- |
| **EXPERIMENTAL DESIGN** |  |  |
| Definition of experimental and control groups | **E** | Longitudinal study with no intervention or control group |
| Number within each group | **E** | Basin Farm soils n= 19  Sewered System are n =20  Non-Sewered area n=32  150-meter buffer area n=10 |
| Assay carried out by core lab or investigator's lab? | D | Investigator's lab |
| **SAMPLE** |  |  |
| Description | **E** | A 10x10x1cm volume of soil was homogenized with a sterile lab scoopula and transferred into three 2ml cryovials and one 5 ml cryovial. |
| Volume/mass of sample processed | D | 200uL |
| Microdissection or macrodissection | **E** | Not applicable |
| Processing procedure | **E** | Initially frozen at -20C on same day as collection. Shipped on dry ice and stored at -80C. |
| Sample storage conditions and duration (especially for FFPE samples) | **E** | Samples were stored at -20C for approximately six months and at -80C for approximately nine months. |
| **NUCLEIC ACID EXTRACTION** |  |  |
| Procedure and/or instrumentation | **E** | See methods section |
| Name of kit and details of any modifications | **E** | Qiagen RNeasy PowerSoil Total RNA kits and then with the Qiagen RNeasy PowerSoil DNA Elution Kit |
| Source of additional reagents used | D | Qiagen Powerbead Pro Tubes |
| Details of DNase or RNAse treatment | **E** | Not applicable |
| Contamination assessment (DNA or RNA) | **E** | At least one extraction negative control was included during each day of extractions |
| Nucleic acid quantification | **E** | Qubit 1X HS dsDNA Kit |
| Instrument and method | **E** | Qubit 4 Fluorometer |
| RNA integrity method/instrument | **E** | Not measured |
| Inhibition testing (Cq dilutions, spike or other) | **E** | Monitored amplification of spiked controls |
| **REVERSE TRANSCRIPTION** |  |  |
| Complete reaction conditions | **E** | One-step reverse transcription |
| Amount of RNA and reaction volume | **E** | Reaction Volume = 1.5 µL |
| Priming oligonucleotide (if using GSP) and concentration | **E** | Proprietary |
| Reverse transcriptase and concentration | **E** | ArrayScript™ Reverse Transcriptase |
| Temperature and time | **E** | 45°C for 20 minutes |
| Manufacturer of reagents and catalogue numbers | D | Applied Biosystems, AgPath-ID™ One-Step RT-PCR Reagents, Catalog number: 4387391 |
| **qPCR TARGET INFORMATION** |  |  |
| If multiplex, efficiency and LOD of each assay. | **E** | Table S1 |
| Location of amplicon | D | See references in Table S1 |
| *In silico* specificity screen (BLAST, etc) | **E** | We BLASTed all assays to confirm specificity before ordering the custom TAC. |
| **qPCR OLIGONUCLEOTIDES** |  |  |
| Primer sequences | **E** | See references in Table S1 |
| Probe sequences | D** | See references in Table S1 |
| Location and identity of any modifications | **E** | No modifications |
| Manufacturer of oligonucleotides | D | ThermoFisher Scientific |
| **qPCR PROTOCOL** |  |  |
| Complete reaction conditions | **E** | 45°C for 20 min and 95°C for 10 min, followed by 45 cycles of 95°C for 15 s and 60°C for 1 min |
| Reaction volume and amount of cDNA/DNA | **E** | 40 µL of template with 60 µL of AgPath-ID™ One-Step RT-PCR Reagents |
| Primer, (probe), Mg++ and dNTP concentrations | **E** | All assays contained the same concentrations of primers (900 nanomolar) and probe (250 nanomolar). The Mg2+ and dNTP concentrations are not listed in the User Guide. |
| Polymerase identity and concentration | **E** | AmpliTaq Gold™ polymerase |
| Buffer/kit identity and manufacturer | **E** | AgPath-ID™ One-Step RT-PCR Reagents |
| Additives (SYBR Green I, DMSO, etc.) | **E** | No additives |
| Manufacturer of plates/tubes and catalog number | D | ThermoFisher Scientific |
| Complete thermocycling parameters | **E** | 45°C for 20 min and 95°C for 10 min, followed by 45 cycles of 95°C for 15 s and 60°C for 1 min |
| Reaction setup (manual/robotic) | D | Manual set-up in a disinfected dead air box (10% bleach with fifteen minutes of contact time, UV for fifteen minutes, and a final cleaning step with 70% ethanol) |
| Manufacturer of qPCR instrument | **E** | ThermoFisher Scientfic |
| **qPCR VALIDATION** |  |  |
| Evidence of optimisation (from gradients) | D | See Liu *et al*. 2013 and Liu *et al*. 2016 |
| Specificity (gel, sequence, melt, or digest) | **E** | See Liu *et al*. 2013 and Liu *et al*. 2016. |
| Standard curves with slope and y-intercept | **E** | See references in Table S2 |
| PCR efficiency calculated from slope | **E** | See references in Table S2 |
| r2 of standard curve | **E** | See references in Table S2 |
| Evidence for limit of detection | **E** | See references in Table S2 |
| **DATA ANALYSIS** |  |  |
| qPCR analysis program (source, version) | **E** | QuantStudio Real-Time PCR Software V1.2 CDC |
| Cq method determination | **E** | Manual thresholding |
| Results of NTCs | **E** | We observed no amplification before at Ct of 40 in our negative controls. However, we did observe unexpected amplification in the *Plasmodium* assay and this was excluded from the manuscript |
| Justification of number and choice of reference genes | **E** |  |
| Description of normalization method | **E** | Volumetric normalization per gram dry soil for soil samples. |
| Software (source, version) | E | R Studio V4.4.0 |

Note: minimum information for publication of quantitative real-time PCR experiments (MIQE)

## S1 Appendix Checklist A. Inclusivity in global research

PLOS’ policy on inclusivity in global research aims to improve transparency in the reporting of research performed outside of researchers’ own country or community and ensures that PLOS publications reporting global research adhere to high standards for research ethics and authorship. Authors of relevant research articles may be asked to complete the questionnaire below, which outlines ethical, cultural, and scientific considerations specific to inclusivity in global research. This questionnaire may be requested when researchers have travelled to a different country to conduct research, if research uses samples collected in another country, research with Indigenous populations or their lands, or if research is on cultural artefacts. Researchers travelling to another country solely to use laboratory equipment will not normally be required to complete the questionnaire. However, the questionnaire can be requested at the journal’s discretion for any submission – if you have been requested to complete this questionnaire by the PLOS journal you submitted to, please do so.

Please complete the questionnaire below and include this as a Supporting Information file with your manuscript. Note that if your paper is accepted for publication, this checklist will be published with your article in the supporting information files. Please ensure that you reference the checklist in the main body of your manuscript. We suggest adding a subsection ‘Inclusivity in global research’ to your Methods section and adding the following sentence: “Additional information regarding the ethical, cultural, and scientific considerations specific to inclusivity in global research is included in the Supporting Information (SX Checklist)”

The questions have been designed to be applicable to a wide range of study types, and there are subsections for both human subjects research and non-human subjects research. If any of the questions are not relevant to your research please mark them as “N/A” as appropriate.

**Ethical considerations, permits and authorship**

*This section is applicable to all research types.*

Provide details as to who granted permissions and/or consent for the study to take place in the Methods section of your manuscript. This should include the names of **all** ethics boards, governmental organizations, community leaders or other bodies that provided approval for the study. If individuals provided approval refer to these people by their role or title but do not list their name(s).

Reported on page number: Page 5 (line 98-100)

If there were any deviations from the study protocol after approval was obtained please provide details of these changes in the Methods section of your manuscript.
Did this study involve local collaborators that are residents of the country where the research was conducted or members of the community studied? If you do not have any authors from said communities, please provide an explanation for this below.

Reported on page number: n/a

Yes, this study involved local collaborators from Maputo Municipal Council and from Centro de Investigação e Treino em Saúde da Polana Caniço (CISPOC), Instituto Nacional de Saúde, Maputo Mozambique.

Everyone listed as an author should meet PLOS’ criteria for authorship and all individuals who meet these criteria should be included in the author byline, rather than the acknowledgements. For further information please see the journal’s Authorship Policy.

**Human subjects research (e.g. health research, medical research, cross-cultural psychology)**

Did you obtain written informed consent from a representative of the local community or region before the research took place? How did you establish who speaks for the community? Details of written informed consent obtained from study participants should be reported separately in the Methods section of your manuscript.

n/a

How did members of the local community provide input on the aims of the research investigation, its methodology, and its anticipated outcome(s)?

n/a

When engaging with the local community, how did you ensure that the informed consent documents and other materials could be understood by local stakeholders?

n/a

Will the findings of the research be made available in an understandable format to stakeholders in the community where the study was conducted (e.g. via a presentation, summary report, copies of publications, etc.)? Please provide details of how this will be achieved.

Copies of publication will be shared with the Maputo Municipal Council.

**Non-human subjects research using specimens/ animals collected as part of the study, or those housed in archival collections. Examples include archaeology, paleontology, botany and zoology.**

Did the permission you obtained from a local authority to perform the study include an agreement on access to outputs and benefit sharing? This may include procedures to enable fair distribution of the benefits and resources arising from the research performed. Please include any details of Prior Informed Consent and Benefit Sharing Agreements obtained. These may be required by field-specific regulations, for example the Convention on Biological Diversity (CBD) and the associated Nagoya Protocol.

The written approval for sampling from the Maputo Municipal Council’s Division of Urban Infrastructure included an agreement for access to outputs in the form of

If the material used in your study was imported, please A) provide the year it was imported and B) indicate whether permits were obtained to import/export the materials used, C) provide details of any permits obtained. If this information is not available, please indicate this.

n/a

If you used archival specimens, please state how the material used in your study was acquired by the institute it is held in and provide details of any permits obtained for the original excavations/ sample collection. If this information is not available, please indicate this.

n/a

How was the potential cultural significance of the materials collected in your study to local communities considered in your research design? Were Indigenous peoples and/or local researchers and institutions involved with archaeological excavations / collection of specimens? If so, please provide a description of their involvement.

n/a

If your manuscript includes photographs of human remains please indicate whether authors obtained permission from descendants or affiliated cultural communities to do so.

n/a

**References**

(1)	Liu, J.; Gratz, J.; Amour, C.; Nshama, R.; Walongo, T.; Maro, A.; Mduma, E.; Platts-Mills, J.; Boisen, N.; Nataro, J.; Haverstick, D. M.; Kabir, F.; Lertsethtakarn, P.; Silapong, S.; Jeamwattanalert, P.; Bodhidatta, L.; Mason, C.; Begum, S.; Haque, R.; Praharaj, I.; Kang, G.; Houpt, E. R. Optimization of Quantitative PCR Methods for Enteropathogen Detection. *PLoS ONE* **2016**, *11* (6), e0158199. https://doi.org/10.1371/journal.pone.0158199.

(2)	Ritalahti, K. M.; Amos, B. K.; Sung, Y.; Wu, Q.; Koenigsberg, S. S.; Löffler, F. E. Quantitative PCR Targeting 16S rRNA and Reductive Dehalogenase Genes Simultaneously Monitors Multiple *Dehalococcoides* Strains. *Appl Environ Microbiol* **2006**, *72* (4), 2765–2774. https://doi.org/10.1128/AEM.72.4.2765-2774.2006.

(3)	Knapp, J.; Umhang, G.; Poulle, M.-L.; Millon, L. Development of a Real-Time PCR for a Sensitive One-Step Coprodiagnosis Allowing Both the Identification of Carnivore Feces and the Detection of Toxocara Spp. and Echinococcus Multilocularis. *Appl Environ Microbiol* **2016**, *82* (10), 2950–2958. https://doi.org/10.1128/AEM.03467-15.

(4)	Centers for Disease Control and Prevention. *Research Use Only 2019-Novel Coronavirus (2019-nCoV) Real-Time RT-PCR Primers and Probes*. https://www.cdc.gov/coronavirus/2019-ncov/lab.

(5)	Ohad, S.; Ben-Dor, S.; Prilusky, J.; Kravitz, V.; Dassa, B.; Chalifa-Caspi, V.; Kashi, Y.; Rorman, E. The Development of a Novel qPCR Assay-Set for Identifying Fecal Contamination Originating from Domestic Fowls and Waterfowl in Israel. *Front. Microbiol.* **2016**, *7*. https://doi.org/10.3389/fmicb.2016.00145.

(6)	Bosman, K. J.; Nijhuis, M.; Van Ham, P. M.; Wensing, A. M. J.; Vervisch, K.; Vandekerckhove, L.; De Spiegelaere, W. Comparison of Digital PCR Platforms and Semi-Nested qPCR as a Tool to Determine the Size of the HIV Reservoir. *Sci Rep* **2015**, *5* (1), 13811. https://doi.org/10.1038/srep13811.

(7)	Bickersmith, S. A.; Lainhart, W.; Moreno, M.; Chu, V. M.; Vinetz, J. M.; Conn, J. E. A Sensitive, Specific and Reproducible Real-Time Polymerase Chain Reaction Method for Detection of Plasmodium vivaxandPlasmodium Falciparum Infection in Field-Collected Anophelines. *Mem. Inst. Oswaldo Cruz* **2015**, *110* (4), 573–576. https://doi.org/10.1590/0074-02760150031.

(8)	Stoddard, R. A.; Gee, J. E.; Wilkins, P. P.; McCaustland, K.; Hoffmaster, A. R. Detection of Pathogenic Leptospira Spp. through TaqMan Polymerase Chain Reaction Targeting the LipL32 Gene. *Diagnostic Microbiology and Infectious Disease* **2009**, *64* (3), 247–255. https://doi.org/10.1016/j.diagmicrobio.2009.03.014.

(9)	Zhuang, F.-F.; Li, H.; Zhou, X.-Y.; Zhu, Y.-G.; Su, J.-Q. Quantitative Detection of Fecal Contamination with Domestic Poultry Feces in Environments in China. *AMB Expr* **2017**, *7* (1), 80. https://doi.org/10.1186/s13568-017-0379-0.

(10)	Zhu, K.; Suttner, B.; Pickering, A.; Konstantinidis, K. T.; Brown, J. A Novel Droplet Digital PCR Human mtDNA Assay for Fecal Source Tracking. *Water Research* **2020**, *183*, 116085. https://doi.org/10.1016/j.watres.2020.116085.

(11)	Barraud, O.; Baclet, M. C.; Denis, F.; Ploy, M. C. Quantitative Multiplex Real-Time PCR for Detecting Class 1, 2 and 3 Integrons. *Journal of Antimicrobial Chemotherapy* **2010**, *65* (8), 1642–1645. https://doi.org/10.1093/jac/dkq167.

(12)	Lanciotti, R. S.; Kosoy, O. L.; Laven, J. J.; Velez, J. O.; Lambert, A. J.; Johnson, A. J.; Stanfield, S. M.; Duffy, M. R. Genetic and Serologic Properties of Zika Virus Associated with an Epidemic, Yap State, Micronesia, 2007. *Emerg. Infect. Dis.* **2008**, *14* (8), 1232–1239. https://doi.org/10.3201/eid1408.080287.

(13)	Tambalo, D. D.; Boa, T.; Liljebjelke, K.; Yost, C. K. Evaluation of Two Quantitative PCR Assays Using Bacteroidales and Mitochondrial DNA Markers for Tracking Dog Fecal Contamination in Waterbodies. *Journal of Microbiological Methods* **2012**, *91* (3), 459–467. https://doi.org/10.1016/j.mimet.2012.09.029.

(14)	Cashdollar, J. L.; Brinkman, N. E.; Griffin, S. M.; McMinn, B. R.; Rhodes, E. R.; Varughese, E. A.; Grimm, A. C.; Parshionikar, S. U.; Wymer, L.; Fout, G. S. Development and Evaluation of EPA Method 1615 for Detection of Enterovirus and Norovirus in Water. *Appl Environ Microbiol* **2013**, *79* (1), 215–223. https://doi.org/10.1128/AEM.02270-12.
