# Supplementary material for: Enteric pathogens in soil adjacent to public waste bins in Maputo, Mozambique offers potential for environmental surveillance
Source: PLOS Glob Public Health. 2026 Aug 18;6(8):e0005998. doi: 10.1371/journal.pgph.0005998 (PMC13485064; doi:10.1371/journal.pgph.0005998)
Supplement: Supplementary file 2 [file pgph.0005998.s003.docx]

| **Comments: Editor/Journal** | **Action Taken** | **Author Response** |
| --- | --- | --- |
| 1. In your Methods section, please provide additional information regarding the permits you obtained for the work. Please ensure you have included the full name of the authority that approved the field site access and, if no permits were required, a brief statement explaining why. | We clarified our approval  We received written approval for sampling from the Maputo Municipal Council’s Division of Urban Infrastructure, which did not require a permit for this work. | Agreed |
| 2. Please include a complete copy of PLOS’ questionnaire on inclusivity in global research in your revised manuscript. Our policy for research in this area aims to improve transparency in the reporting of research performed outside of researchers’ own country or community. The policy applies to researchers who have travelled to a different country to conduct research, research with Indigenous populations or their lands, and research on cultural artefacts. The questionnaire can also be requested at the journal’s discretion for any other submissions, even if these conditions are not met.  Please find more information on the policy and a link to download a blank copy of the questionnaire here: <https://journals.plos.org/globalpublichealth/s/best-practices-in-research-reporting>. Please upload a completed version of your questionnaire as Supporting Information when you resubmit your manuscript. | We have included a complete copy of PLOS’ questionnaire on inclusivity in global research in our revised manuscript in the SI. | Agreed |
| 3. Please provide a detailed online Financial Disclosure statement. This is published with the article. It must therefore be completed in full sentences and contain the exact wording you wish to be published.  a) State the initials, alongside each funding source, of each author to receive each grant. For example: “This work was supported by the National Institutes of Health (####### to AM; ###### to CJ) and the National Science Foundation (###### to AM).”  b) State what role the funders took in the study. If the funders had no role in your study, please state: “The funders had no role in study design, data collection and analysis, decision to publish, or preparation of the manuscript.”  For more information, please go to our submission guidelines: https://journals.plos.org/globalpublichealth/s/submission-guidelines#loc-financial-disclosure-statement | Revised the financial disclosure statement. |  |
| 4. Please ensure that the funders and grant numbers match between the Financial Disclosure field and the Funding Information tab in your submission form. Note that the funders must be provided in the same order in both places as well. | Updated these boxes in the portal. |  |
| 5. Please update your online Competing Interests statement. If you have no competing interests to declare, please state: "The authors have declared that no competing interests exist." | Updated the competing interests statement. |  |
| 6. Please provide separate main figure files in .tif or .eps format only and remove any figures embedded in your manuscript file. Please also ensure that all files are under our size limit of 10MB. Please leave the figure captions in the manuscript.  For more information about how to convert your figure files please see our guidelines: <https://journals.plos.org/globalpublichealth/s/figures> | These files have been switched to .tif format, and have been removed from the manuscript file. | Agreed |
| 7. Please ensure that you refer to Figure 3 in your text as, if accepted, production will need this reference to link the reader to the figure. | Thank you for the comment, this has been fixed. | Agreed |
| 8. We notice that your supplementary tables are included in the manuscript file. Please remove them and upload them with the file type 'Supporting Information'. Please ensure that each Supporting Information file has a legend listed in the manuscript before or after the references list. | Thank you for the comment, all SI material has been moved to the supporting information file. | Agreed |
| 9. Some material included in your submission may be copyrighted. According to PLOS’s copyright policy, authors who use figures or other material (e.g., graphics, clipart, maps) from another author or copyright holder must demonstrate or obtain permission to publish this material under the Creative Commons Attribution 4.0 International (CC BY 4.0) License used by PLOS journals. Please closely review the details of PLOS’s copyright requirements here: PLOS Licenses and Copyright. If you need to request permissions from a copyright holder, you may use PLOS's Copyright Content Permission form.  Please respond directly to this email or email the journal office and provide any known details concerning your material's license terms and permissions required for reuse, even if you have not yet obtained copyright permissions or are unsure of your material's copyright compatibility.  Potential Copyright Issues:  Figure 3: please (a) provide a direct link to the base layer of the map (i.e., the country or region border shape) and ensure this is also included in the figure legend; and (b) provide a link to the terms of use / license information for the base layer image or shapefile. We cannot publish proprietary or copyrighted maps (e.g. Google Maps, Mapquest) and the terms of use for your map base layer must be compatible with our CC-BY 4.0 license.  Note: if you created the map in a software program like R or ArcGIS, please locate and indicate the source of the basemap shapefile onto which data has been plotted.  If your map was obtained from a copyrighted source please amend the figure so that the base map used is from an openly available source. Alternatively, please provide explicit written permission from the copyright holder granting you the right to publish the material under our CC-BY 4.0 license.  Please note that the following CC BY licenses are compatible with PLOS license: CC BY 4.0, CC BY 2.0 and CC BY 3.0, meanwhile such licenses as CC BY-ND 3.0 and others are not compatible due to additional restrictions.  If you are unsure whether you can use a map or not, please do reach out and we will be able to help you. The following websites are good examples of where you can source open access or public domain maps:  * U.S. Geological Survey (USGS) - All maps are in the public domain. ([http://www.usgs.gov](http://www.usgs.gov/))  * PlaniGlobe - All maps are published under a Creative Commons license so please cite “PlaniGlobe, [http://www.planiglobe.com](http://www.planiglobe.com/), CC BY 2.0” in the image credit after the caption. (<http://www.planiglobe.com/?lang=enl>)  * Natural Earth - All maps are public domain. (<http://www.naturalearthdata.com/about/terms-of-use/>) | We have updated the basemap to be open street maps: and have added the following to the figure caption.  © OpenStreetMap contributors (2024). OpenStreetMap. https://www.openstreetmap.org | Agreed |
| 10. If the reviewer comments include a recommendation to cite specific previously published works, please review and evaluate these publications to determine whether they are relevant and should be cited. There is no requirement to cite these works unless the editor has indicated otherwise. | We have reviewed and evaluated publications and concluded they were relevant and should be cited. | Thank you for your comment. |
| 11. Please review your reference list to ensure that it is complete and correct. If you have cited papers that have been retracted, please include the rationale for doing so in the manuscript text, or remove these references and replace them with relevant current references. Any changes to the reference list should be mentioned in the rebuttal letter that accompanies your revised manuscript. If you need to cite a retracted article, indicate the article’s retracted status in the References list and also include a citation and full reference for the retraction notice. | Reviewed reference list | Thank you for your comment, we have reviewed our reference list and believe it is up to date with not retracted papers. |
| **Reviewer # 1 Comments** |  |  |
| I think that in the Introduction, the authors need to acknowledge existing work from LMICs related to wastewater surveillance in non-sewered sanitation settings. There is considerable global work in this area. For example (Line 55–56), the authors could cite studies such as: “COVID-19 monitoring with sparse sampling of sewered and non-sewered wastewater in urban and rural communities.” iScience 26.7 (2023). “Global participatory wastewater surveillance to understand mpox clade diversity in war and conflict-affected countries.” Journal of Travel Medicine 32.5 (2025): taaf052. “Wastewater-based epidemiological surveillance of SARS-CoV-2 new variants BA.2.86 and offspring JN.1 in South and Southeast Asia.” Journal of Travel Medicine 31.4 (2024): taae040. | Citations have been added to support our claim and acknowledge existing work. | Thank you for your comments, citations have been updated to improve this section. Line 55 |
| In the Results section, HIV was detected only in the Basin farm area and not in the other locations. This is an interesting finding. Have the authors explored possible reasons for this and considered investigating it further? Similarly, the higher detection of norovirus and SARS-CoV-2 in this location is intriguing. I wonder whether this discrepancy could be related to the lower human mtDNA levels observed in this area compared with other sites. |  | Thank you for your comment. While there was higher detection of HIV in the basin farming area and a higher detection of SARS-CoV-2, these were not statistically significant differences between the other sampling locations. While interesting, this was outside the scope of the study. |
| It is also interesting that influenza or avian influenza were not detected in any of the samples. Is there a specific reason for this? |  | Thank you for your comment, We did not test for influenza or avian influenza. Using TaqMan array cards, there is a maximum number of targets that we can tests, and these were not chosen. While interesting, this was outside the scope of the study. |
| Finally, I am also wondering whether wastewater is used for farming or irrigation in the study areas, or whether contaminated waste is reused as a type of compost. This could potentially influence the environmental distribution of pathogens. | “Subsistence farm plots were used for comparative purposes due to their proximity to the WWTP and potential irrigation with partially treated wastewater.” Line 107-109 | Thank you for your comment. |
| **Reviewer #2 Comments** |  |  |
| Line 97: The authors acknowledge convenience sampling but the limitations of the approach should be discussed in detail. In particular, the 150m buffer zone the authors talk about has only 10 samples but this is the key finding with highest count and aar. Author should acknowledge these results are subject to uncertainties. Authors should be cautious with this in discussion. | The following lines has been added to the results section to address this comment:  “Our sample size was also relatively small, and some sub-groups (e.g., soils in the buffer area) contained few samples. Additional study with a larger sample size would be useful to assess the impact of seasonality or rainfall events on enteric pathogen detection in soils.” Line 336-339 | Thank you for your comment. This is a good point. We have updated the discussion section to include a statement discussing this. |
| Line 112: The authors should describe how the 150m buffer zone was decided, if it is infrastructurally meaningful and if it has been used in the past citing previous studies. | The following line has been added to address this comment: These boundary roads represent the main roads of access between the sewered or non-sewered areas and serve as the main roads for access to the central business district, Maputo port, and other key urban infrastructure destinations. Line 116-119 | Thank you for your comment. The buffer zone was decided based off of the rounded-up distance of 2 city-block on average. This distance was chosen based on likely walking paths in relation to the dividing line between sewered and non-sewered areas on highly trafficked roads that observed are the main avenues of traffic within Maputo. |
| Line 163: Provide proper citation | Citation has been added. | Citation for r Version 4.4.1 has been added. |
| Line 163: Cq cutoff of 35 is fine but I would suggest providing some citations here. | Citation has been added.  Platts-Mills JA, Gratz J, Mduma E, Svensen E, Amour C, Liu J, Maro A, Saidi Q, Swai N, Kumburu H, McCormick BJ, Kibiki G, Houpt ER. Association between stool enteropathogen quantity and disease in Tanzanian children using TaqMan array cards: a nested case-control study. Am J Trop Med Hyg. 2014 Jan;90(1):133-8. doi: 10.4269/ajtmh.13-0439. Epub 2013 Nov 4. PMID: 24189366; PMCID: PMC3886409. | Thank you for your comment. We have provided a citation for the Cq cutoff. |
| Line 166: The paper does not report any model diagnostics or discuss whether the assumptions of the model were assessed for Poisson regression. Given the variability in some of the pathogen count data, the authors should report a dispersion statistic and briefly address potential overdispersion. | Table 2 has been updated to reflect change from Poisson regression to quasi-Poisson regression.  The following line has been added to the methods section: Overdispersion was identified in the Poisson models (dispersion statistic > 2), and quasi-Poisson regressions were applied to account for this and ensure appropriate variance estimation. Line 170-171 | Thank you for bringing this up, we have run a dispersion statistic and did identify overdispersion in the data set. We have used a quasi-regression to address this dispersion. The results of the new model have not changed the findings. |
| Line 172: The storm drain area is what? Is it one of the four sampling locations? If it is non-sewered area, please be consistent. | Thank you for catching this, storm drain area is the non-sewered area. This has been updated to ‘non-sewered area’. Line 176 | Thank you for your comment, this has been changed. |
| Line 206: Could low viral detection attributed to RNA degradation in soil or limitation in RNA extraction step. If authors believe so, this should be addressed. | Clarified sample storage:  Soil samples were stored at -20°C for up to six months and then were shipped on dry ice (-80°C) with temperature monitoring to the University of North Carolina at Chapel Hill, North Carolina for molecular analysis. Line 134-136  The following line has been added to the limitations section: In addition, samples were stored at -20^o^C, which may have resulted in some nucleic acid degradation, which may have prevented us from detecting some targets. 335-336 | Thank you for your comment. |
| Line 179: Can the authors overlay individual data points overlaid on the boxes so the actual spread of the data for small sample size be seen. | Data points have been added to Figure 1 to show spread. | Thank you for your comment. This has been added. Points below the box-and-whisker are 0 values (which are -inf in log plots). |
| Line 185: Is it Mycobacterium tuberculosis or Mycoplasma? | This has been corrected to Mycobacterium tuberculosis. | Thank you for catching this naming mistake. |
| Line 216: Replace system with sampling location or something similar. Please overlay data points. | The x-axis has been updated, with data points added for Figure 2. | Thank you for your comment, this has been changed. |
| Throughout the manuscript: E. coli (C is capitalized in many places) | All instances have been fixed to E. coli | Thank you for your comment. |
