# Supplementary material for: Enteric pathogens in soil adjacent to public waste bins in Maputo, Mozambique offers potential for environmental surveillance
Source: PLOS Glob Public Health. 2026 Aug 18;6(8):e0005998. doi: 10.1371/journal.pgph.0005998 (PMC13485064; doi:10.1371/journal.pgph.0005998)
Supplement: Peer review history

# Peer review: Decision Letter 0

**DOI:** 10.1371/journal.pgph.0005998.r001

**Authors:** Mohan Amarasiri

22 Jun 2026

PGPH-D-26-00394

Enteric Pathogens in Soil Adjacent to Public Waste Bins in Maputo, Mozambique Offers Potential for Environmental Surveillance

PLOS Global Public Health

Dear Dr. Capone,

Thank you for submitting your manuscript to PLOS Global Public Health. After careful consideration, we feel that it has merit but does not fully meet PLOS Global Public Health’s publication criteria as it currently stands. Therefore, we invite you to submit a revised version of the manuscript that addresses the points raised during the review process.

Both reviewers have identified the importance of the study and have provided minor comments.

Please submit your revised manuscript by Jul 22 2026 11:59PM. If you will need more time than this to complete your revisions, please reply to this message or contact the journal office at globalpubhealth@plos.org. When you're ready to submit your revision, log on to https://www.editorialmanager.com/pgph/ and select the 'Submissions Needing Revision' folder to locate your manuscript file.

Please include the following items when submitting your revised manuscript:

- A letter that responds to each point raised by the editor and reviewer(s). You should upload this letter as a separate file labeled 'Response to Reviewers'.
- A marked-up copy of your manuscript that highlights changes made to the original version. You should upload this as a separate file labeled 'Revised Manuscript with Track Changes'.
- An unmarked version of your revised paper without tracked changes. You should upload this as a separate file labeled 'Manuscript'.

Guidelines for resubmitting your figure files are available below the reviewer comments at the end of this letter.

As the corresponding author, your ORCID iD is verified in the submission system and will appear in the published article. PLOS supports the use of ORCID, and we encourage all coauthors to register for an ORCID iD and use it as well. Please encourage your coauthors to verify their ORCID iD within the submission system before final acceptance, as unverified ORCID iDs will not appear in the published article. *Only*  the individual author can complete the verification step; PLOS staff *cannot*  verify ORCID iDs on behalf of authors.

We look forward to receiving your revised manuscript.

Kind regards,

Mohan Amarasiri

Academic Editor

PLOS Global Public Health

Journal Requirements:

1. In your Methods section, please provide additional information regarding the permits you obtained for the work. Please ensure you have included the full name of the authority that approved the field site access and, if no permits were required, a brief statement explaining why.

2. Please include a complete copy of PLOS’ questionnaire on inclusivity in global research in your revised manuscript. Our policy for research in this area aims to improve transparency in the reporting of research performed outside of researchers’ own country or community. The policy applies to researchers who have travelled to a different country to conduct research, research with Indigenous populations or their lands, and research on cultural artefacts. The questionnaire can also be requested at the journal’s discretion for any other submissions, even if these conditions are not met.  Please find more information on the policy and a link to download a blank copy of the questionnaire here: https://journals.plos.org/globalpublichealth/s/best-practices-in-research-reporting. Please upload a completed version of your questionnaire as Supporting Information when you resubmit your manuscript.

3. Please provide a detailed online Financial Disclosure statement. This is published with the article. It must therefore be completed in full sentences and contain the exact wording you wish to be published.

a) State the initials, alongside each funding source, of each author to receive each grant. For example: “This work was supported by the National Institutes of Health (####### to AM; ###### to CJ) and the National Science Foundation (###### to AM).”

b) State what role the funders took in the study. If the funders had no role in your study, please state: “The funders had no role in study design, data collection and analysis, decision to publish, or preparation of the manuscript.”

For more information, please go to our submission guidelines:

https://journals.plos.org/globalpublichealth/s/submission-guidelines#loc-financial-disclosure-statement

4. Please ensure that the funders and grant numbers match between the Financial Disclosure field and the Funding Information tab in your submission form. Note that the funders must be provided in the same order in both places as well.

5. Please update your online Competing Interests statement. If you have no competing interests to declare, please state: "The authors have declared that no competing interests exist."

6. Please provide separate main figure files in .tif or .eps format only and remove any figures embedded in your manuscript file. Please also ensure that all files are under our size limit of 10MB. Please leave the figure captions in the manuscript.

For more information about how to convert your figure files please see our guidelines: https://journals.plos.org/globalpublichealth/s/figures

7. Please ensure that you refer to Figure 3 in your text as, if accepted, production will need this reference to link the reader to the figure.

8. We notice that your supplementary tables are included in the manuscript file. Please remove them and upload them with the file type 'Supporting Information'. Please ensure that each Supporting Information file has a legend listed in the manuscript before or after the references list.

9. Some material included in your submission may be copyrighted. According to PLOS’s copyright policy, authors who use figures or other material (e.g., graphics, clipart, maps) from another author or copyright holder must demonstrate or obtain permission to publish this material under the Creative Commons Attribution 4.0 International (CC BY 4.0) License used by PLOS journals. Please closely review the details of PLOS’s copyright requirements here: PLOS Licenses and Copyright. If you need to request permissions from a copyright holder, you may use PLOS's Copyright Content Permission form.

Please respond directly to this email or email the journal office and provide any known details concerning your material's license terms and permissions required for reuse, even if you have not yet obtained copyright permissions or are unsure of your material's copyright compatibility.

Potential Copyright Issues:

Figure 3: please (a) provide a direct link to the base layer of the map (i.e., the country or region border shape) and ensure this is also included in the figure legend; and (b) provide a link to the terms of use / license information for the base layer image or shapefile. We cannot publish proprietary or copyrighted maps (e.g. Google Maps, Mapquest) and the terms of use for your map base layer must be compatible with our CC-BY 4.0 license.

Note: if you created the map in a software program like R or ArcGIS, please locate and indicate the source of the basemap shapefile onto which data has been plotted.

If your map was obtained from a copyrighted source please amend the figure so that the base map used is from an openly available source. Alternatively, please provide explicit written permission from the copyright holder granting you the right to publish the material under our CC-BY 4.0 license.

Please note that the following CC BY licenses are compatible with PLOS license: CC BY 4.0, CC BY 2.0 and CC BY 3.0, meanwhile such licenses as CC BY-ND 3.0 and others are not compatible due to additional restrictions.

If you are unsure whether you can use a map or not, please do reach out and we will be able to help you. The following websites are good examples of where you can source open access or public domain maps:

* U.S. Geological Survey (USGS) - All maps are in the public domain. (http://www.usgs.gov)

* PlaniGlobe - All maps are published under a Creative Commons license so please cite “PlaniGlobe, http://www.planiglobe.com, CC BY 2.0” in the image credit after the caption. (http://www.planiglobe.com/?lang=enl)

* Natural Earth - All maps are public domain. (http://www.naturalearthdata.com/about/terms-of-use/)

10. If the reviewer comments include a recommendation to cite specific previously published works, please review and evaluate these publications to determine whether they are relevant and should be cited. There is no requirement to cite these works unless the editor has indicated otherwise.

11. Please review your reference list to ensure that it is complete and correct. If you have cited papers that have been retracted, please include the rationale for doing so in the manuscript text, or remove these references and replace them with relevant current references. Any changes to the reference list should be mentioned in the rebuttal letter that accompanies your revised manuscript. If you need to cite a retracted article, indicate the article’s retracted status in the References list and also include a citation and full reference for the retraction notice.

Additional Editor Comments (if provided):

[Note: HTML markup is below. Please do not edit.]

Reviewers' comments:

Reviewer's Responses to Questions

**Comments to the Author**

1. Does this manuscript meet PLOS Global Public Health’s publication criteria?

Reviewer #1: Yes

Reviewer #2: Yes

**********

2. Has the statistical analysis been performed appropriately and rigorously?

Reviewer #1: Yes

Reviewer #2: Yes

**********

3. Have the authors made all data underlying the findings in their manuscript fully available (please refer to the Data Availability Statement at the start of the manuscript PDF file)?

The PLOS Data policy

Reviewer #1: Yes

Reviewer #2: Yes

**********

4. Is the manuscript presented in an intelligible fashion and written in standard English?

Reviewer #1: Yes

Reviewer #2: Yes

**********

Reviewer #1: This is an interesting piece of work, especially in LMIC settings, and it provides valuable results that are important for other LMICs as well.

I think that in the Introduction, the authors need to acknowledge existing work from LMICs related to wastewater surveillance in non-sewered sanitation settings. There is considerable global work in this area. For example (Line 55–56), the authors could cite studies such as: “COVID-19 monitoring with sparse sampling of sewered and non-sewered wastewater in urban and rural communities.” iScience 26.7 (2023). “Global participatory wastewater surveillance to understand mpox clade diversity in war and conflict-affected countries.” Journal of Travel Medicine 32.5 (2025): taaf052. “Wastewater-based epidemiological surveillance of SARS-CoV-2 new variants BA.2.86 and offspring JN.1 in South and Southeast Asia.” Journal of Travel Medicine 31.4 (2024): taae040.

In the Results section, HIV was detected only in the Basin farm area and not in the other locations. This is an interesting finding. Have the authors explored possible reasons for this and considered investigating it further? Similarly, the higher detection of norovirus and SARS-CoV-2 in this location is intriguing. I wonder whether this discrepancy could be related to the lower human mtDNA levels observed in this area compared with other sites.

It is also interesting that influenza or avian influenza were not detected in any of the samples. Is there a specific reason for this?

Finally, I am also wondering whether wastewater is used for farming or irrigation in the study areas, or whether contaminated waste is reused as a type of compost. This could potentially influence the environmental distribution of pathogens.

Reviewer #2: General comments:

This paper tackles an important gap in the environmental surveillance literature, how do we extend wastewater-based epidemiology (WBE) to settings that don't have centralized sewer infrastructure? The central idea of using soils adjacent to public waste bins as a proxy matrix for enteric pathogen monitoring is practically motivated, and the Maputo context makes it an interesting case study given the well-described sanitation divide between the sewered and non-sewered districts. The research question is well-defined, the pathogen panel is comprehensive, the inclusion of fecal source tracking markers adds depth to the interpretation. The paper also does a reasonable job situating itself within both the WBE and WASH literature. That said, there are several concerns about study design, analytical transparency, and interpretive overreach that should be addressed before publication.

The main issues I see on the paper are: (a) convenience sampling is used without the resulting limits on sample representativeness (b) the Poisson regression analysis is not reported in sufficient detail, with no discussion of model diagnostics, overdispersion, or goodness-of-fit (c) some conclusions in the Discussion extend beyond what the data support and (d) several presentation and consistency issues should be corrected.

Specific comments:

Line 97: The authors acknowledge convenience sampling but the limitations of the approach should be discussed in detail. In particular, the 150m buffer zone the authors talk about has only 10 samples but this is the key finding with highest count and aar. Author should acknowledge these results are subject to uncertainties. Authors should be cautious with this in discussion.

Line 112: The authors should describe how the 150m buffer zone was decided, if it is infrastructurally meaningful and if it has been used in the past citing previous studies.

Line 163: Provide proper citation

Line 163: Cq cutoff of 35 is fine but I would suggest providing some citations here.

Line 166: The paper does not report any model diagnostics or discuss whether the assumptions of the model were assessed for Poisson regression. Given the variability in some of the pathogen count data, the authors should report a dispersion statistic and briefly address potential overdispersion.

Line 172: The storm drain area is what? Is it one of the four sampling locations? If it is non-sewered area, please be consistent.

Line 206: Could low viral detection attributed to RNA degradation in soil or limitation in RNA extraction step. If authors believe so, this should be addressed.

Line 179: Can the authors overlay individual data points overlaid on the boxes so the actual spread of the data for small sample size be seen.

Line 185: Is it Mycobacterium tuberculosis or Mycoplasma?

Line 216: Replace system with sampling location or something similar. Please overlay data points.

Throughout the manuscript:

E. coli (C is capitalized in many places)

**********

what does this mean?). If published, this will include your full peer review and any attached files.

**Do you want your identity to be public for this peer review?**  If you choose “no”, your identity will remain anonymous but your review may still be made public.

For information about this choice, including consent withdrawal, please see our Privacy Policy

Reviewer #1: No

Reviewer #2: No

**********

[NOTE: If reviewer comments were submitted as an attachment file, they will be attached to this email and accessible via the submission site. Please log into your account, locate the manuscript record, and check for the action link "View Attachments". If this link does not appear, there are no attachment files.]

# Author comment: Author response to Decision Letter 1

**DOI:** 10.1371/journal.pgph.0005998.r002

16 Jul 2026

Attachment Submitted filename: Dalton_et_al_Enteric_Pathogens_Public_Waste_Comments_JD_3.docx

# Editor report: Decision Letter 1

**DOI:** 10.1371/journal.pgph.0005998.r003

**Authors:** Mohan Amarasiri

22 Jul 2026

Enteric Pathogens in Soil Adjacent to Public Waste Bins in Maputo, Mozambique Offers Potential for Environmental Surveillance

PGPH-D-26-00394R1

Dear Dr. Capone,

We are pleased to inform you that your manuscript 'Enteric Pathogens in Soil Adjacent to Public Waste Bins in Maputo, Mozambique Offers Potential for Environmental Surveillance' has been provisionally accepted for publication in PLOS Global Public Health.

Before your manuscript can be formally accepted you will need to complete some formatting changes, which you will receive in a follow up email. A member of our team will be in touch with a set of requests.

Please note that your manuscript will not be scheduled for publication until you have made the required changes, so a swift response is appreciated.

IMPORTANT: The editorial review process is now complete. PLOS will only permit corrections to spelling, formatting or significant scientific errors from this point onwards. Requests for major changes, or any which affect the scientific understanding of your work, will cause delays to the publication date of your manuscript.

If your institution or institutions have a press office, please notify them about your upcoming paper to help maximize its impact. If they'll be preparing press materials, please inform our press team as soon as possible -- no later than 48 hours after receiving the formal acceptance. Your manuscript will remain under strict press embargo until 2 pm Eastern Time on the date of publication. For more information, please contact globalpubhealth@plos.org.

Thank you again for supporting Open Access publishing; we are looking forward to publishing your work in PLOS Global Public Health.

Best regards,

Mohan Amarasiri

Academic Editor

PLOS Global Public Health

***********************************************************

Please complete the names of bacteria such as Aeromonas, Vibrio in the proof reading step.

Reviewer Comments (if any, and for reference):
